# Supplementary material for: DNA damage alters nuclear mechanics through chromatin reorganization
Source: Nucleic Acids Res. 2020 Dec 16;49(1):340–53. doi: 10.1093/nar/gkaa1202 (PMC7797048; doi:10.1093/nar/gkaa1202)
Supplement: gkaa1202_Supplemental_File [file gkaa1202_supplemental_file.pdf]

DNA damage alters nuclear mechanics through chromatin reorganisation

Ália dos Santos[1], Alexander W. Cook[1], Rosemarie E Gough[1], Martin Schilling[2], Nora Aleida Olszok[2], Ian Brown[3], Lin Wang[4], Jesse Aaron[5], Marisa L. Martin-Fernandez[4], Florian Rehfeldt[2,6]* and Christopher P. Toseland[1]*

Supplementary Figures

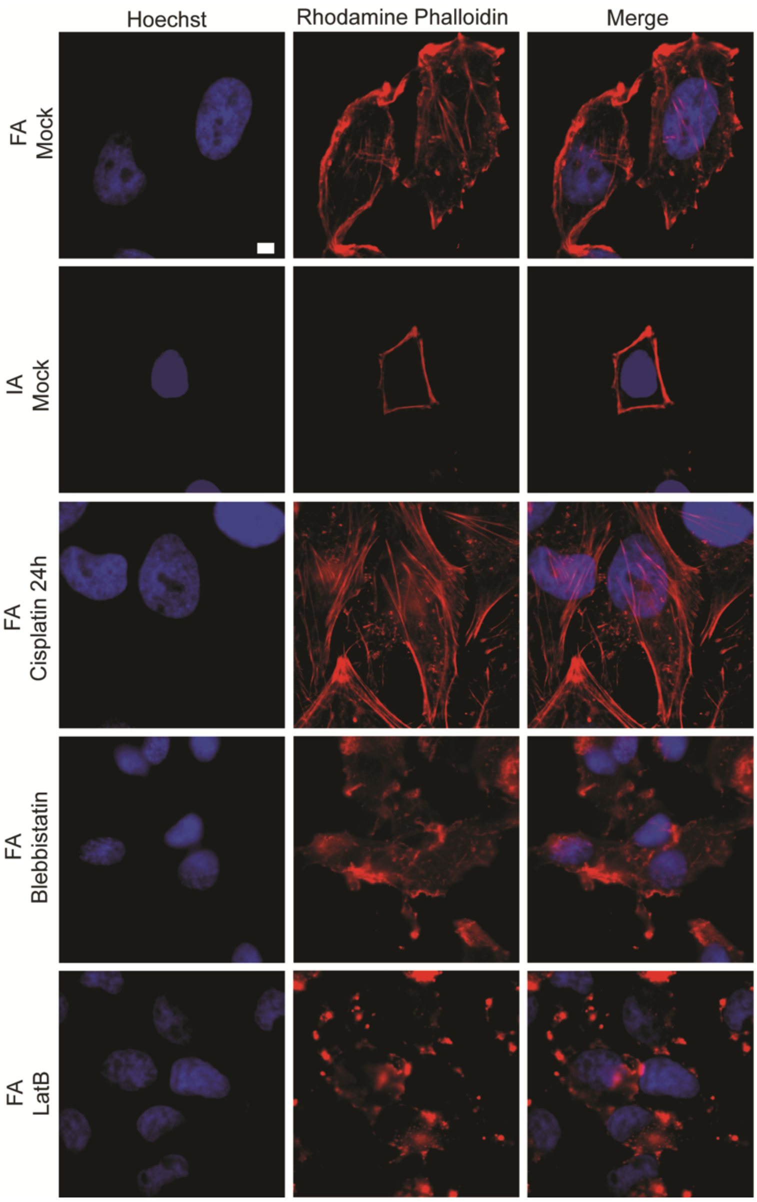

Supplementary Figure 1 – Actin labelling of HeLa cells under different conditions. Wide-field microscopy of actin labelled with Rhodamine Phalloidin, shown in red, and nuclear stain Hoechst, in blue, under stated conditions. (Scale bar = 5µm).

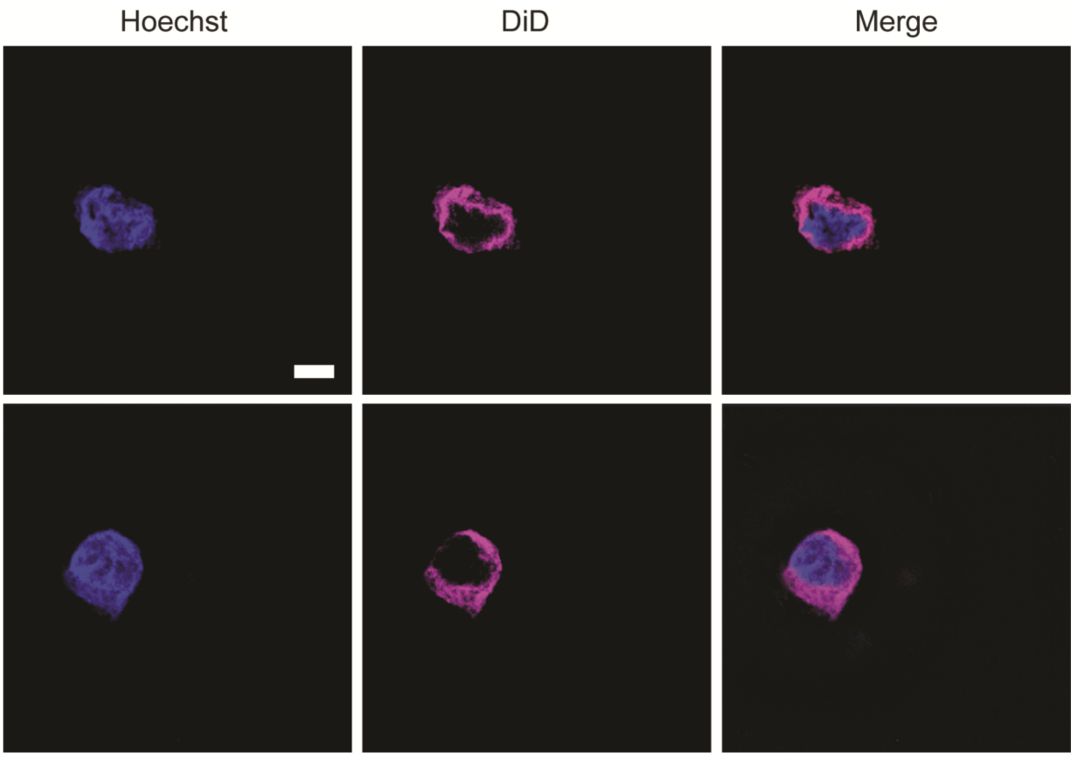

Supplementary Figure 2 – Membrane integrity in isolated nuclei. Wide-Field image of isolated nuclei with Hoechst DNA labelling and membrane staining using DiD. (Scale bar = 5μm).

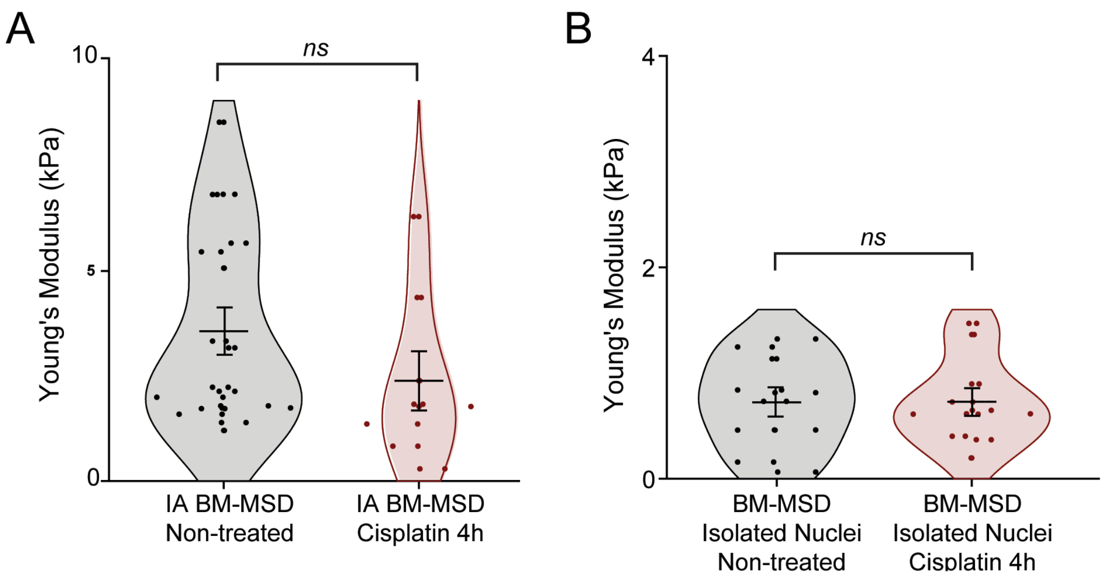

Supplementary Figure 3 – Mechanics of Bone-marrow Mesenchymal stem cells (BM-MSD). (A) Young's modulus values for initially adhered BM-MSD cells before and after 4h cisplatin treatment. Non-treated: n= 17, mean +/- SE = 3.5 +/- 0.6. Cisplatin 4h: n = 8, mean +/- SE = 2.4 +/- 0.7. p = 0.23. (B) Young's modulus values for BM-MSD nuclei from non-treated cells and following 4h cisplatin treatment. Non-treated: n= 10, mean +/- SE = 0.7 +/- 0.1. Cisplatin 4h: n = 10, mean +/- SE = 0.7 +/- 0.1. p = 0.97. (B)

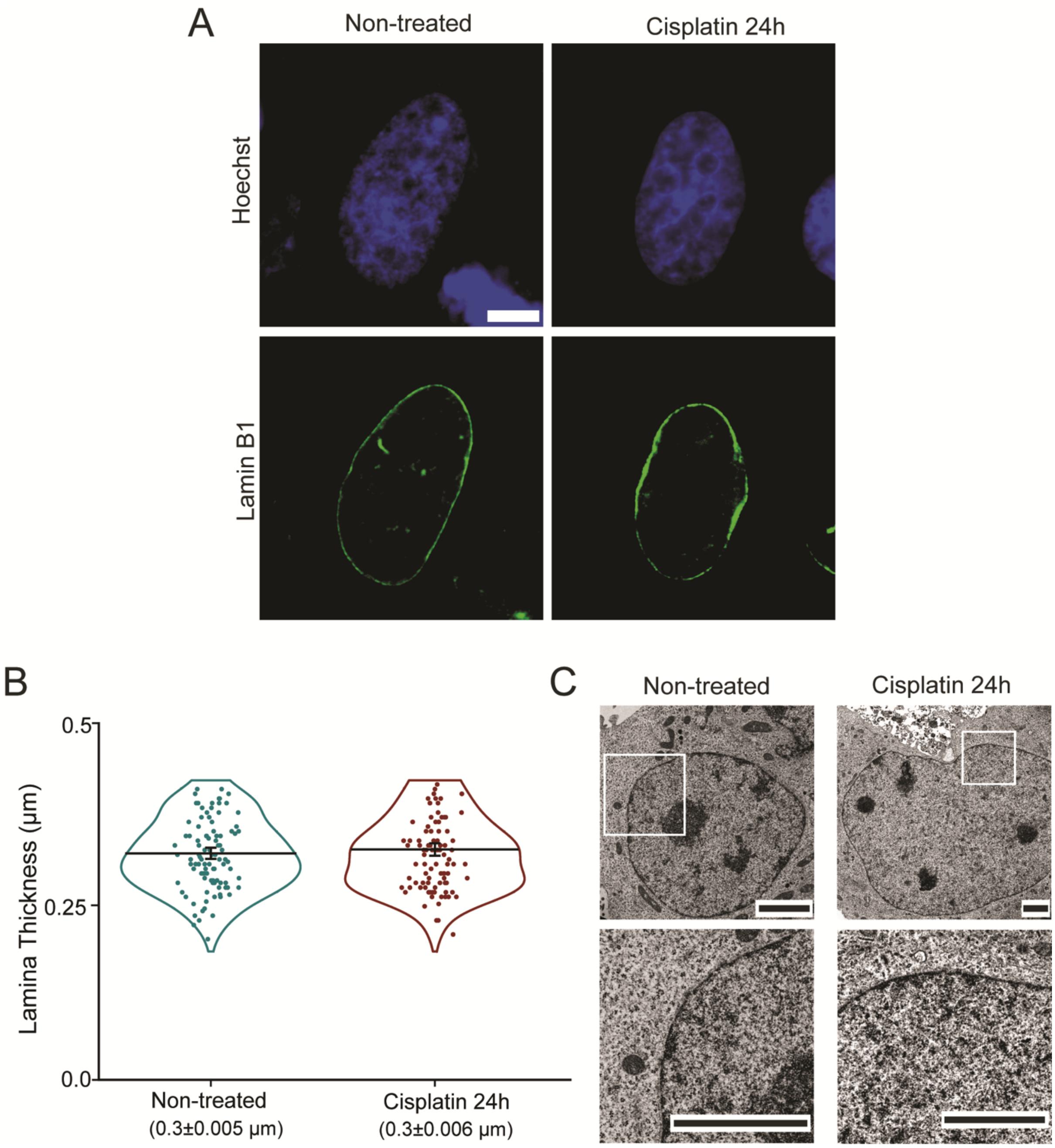

Supplementary Figure 4 – Nuclear envelope integrity in HeLa cells after cisplatin treatment. (A) Wide-field immunofluorescent images of Lamin B1 in non-treated and cisplatin-treated cells (green). (Scale bar = 5µm). **(B)** Measurements of nuclear lamina thickness, using Lamin B1 as a marker. Each point represents the average of 5 measurements at different regions in the nucleus of non-treated (n = *101*) and long cisplatin treatment (n = *100*) $p > 0.05$, calculated using a two-tailed t-test and assuming equal variance*.* Mean ± SE values are plotted. **(C)** Electron microscopy images showing membrane integrity in nuclei of both conditions. Lower panel shows zoomed in region in white square. (Scale bar = 2µm).
